# The prognostic impact of programmed cell death ligand 1 and human leukocyte antigen class I in pancreatic cancer

**DOI:** 10.1002/cam4.1087

**Published:** 2017-06-10

**Authors:** Daisuke Imai, Tomoharu Yoshizumi, Shinji Okano, Hideaki Uchiyama, Toru Ikegami, Norifumi Harimoto, Shinji Itoh, Yuji Soejima, Shinichi Aishima, Yoshinao Oda, Yoshihiko Maehara

**Affiliations:** ^1^ Department of Surgery and Science Graduate School of Medical Sciences Kyushu University Fukuoka Japan; ^2^ Department of General surgery Digestive Disease and Surgery institute Cleveland Clinic Cleveland USA; ^3^ Department of Pathology and Microbiology Saga Medical School Faculty of Medicine Saga University Saga Japan; ^4^ Department of Anatomic Pathology Pathological sciences Graduate School of Medical Sciences Kyushu University Fukuoka Japan

**Keywords:** Biomarker, human leukocyte antigens class I, immunotherapy, pancreatic cancer, programmed cell death ligand 1

## Abstract

Pancreatic ductal adenocarcinoma (PDA) is associated with an immunosuppressive tumor‐microenvironment (TME) that supports the growth of tumors and mediates tumors enabling evasion of the immune system. Expression of programmed cell death ligand 1 (PD‐L1) and loss of human leukocyte antigen (HLA) class I on tumor cells are methods by which tumors escape immunosurveillance. We examined immune cell infiltration, the expression of PD‐L1 and HLA class I by PDA cells, and the correlation between these immunological factors and clinical prognosis. PDA samples from 36 patients were analyzed for HLA class I, HLA‐DR, PD‐L1, PD‐1, CD4, CD8, CD56, CD68, and FoxP3 expression by immunohistochemistry. The correlations between the expression of HLA class I, HLA‐DR, PD‐L1 or PD‐1 and the pattern of tumor infiltrating immune cells or the patients’ prognosis were assessed. PD‐L1 expression correlated with tumor infiltration by CD68^+^ and FoxP3^+^ cells. Low HLA class I expression was an only risk factor for poor survival. PD‐L1 negative and HLA class I high‐expressing PDA was significantly associated with higher numbers of infiltrating CD8^+^ T cells in the TME, and a better prognosis. Evaluation of both PD‐L1 and HLA class I expression by PDA may be a good predictor of prognosis for patients. HLA class I expression by tumor cells should be evaluated when selecting PDA patients who may be eligible for treatment with PD‐1/PD‐L1 immune checkpoint blockade therapies.

## Introduction

Pancreatic ductal adenocarcinoma (PDA) is one of the most lethal human malignancies and the fourth leading cause of mortality in Japan 1. Current therapies remain minimally effective at treating late‐stage disease. Cancer immunotherapy has recently made progress by focusing on overcoming T‐cell regulatory checkpoints with blocking monoclonal antibodies directed against cytotoxic T‐lymphocyte associated protein‐4 or programmed cell death 1/programmed cell death ligand 1 (PD‐1/PD‐L1) 2. Antibody therapy has a potential to become a standard treatment for many cancer types, including PDA. However, attempts to treat PDA with immunotherapies has achieved only limited efficacy 3, 4.

PDA is characterized by the presence of a dense desmoplastic stroma infiltrated with immunosuppressive myeloid‐derived suppressor cells, macrophages, fibroblasts, and regulatory T cells (T_reg_) 5, 6, 7, 8, 9. The immunosuppressive tumor microenvironment (TME) often does not contain effector T cells and those that do reach TME are subsequently inactivated 10, 11. The lack of response to immune checkpoint blockade therapies by PDA patients, as well as colorectal cancer, ovarian cancer and prostate cancer patients, has been proposed to be a result of the establishment of an “immune privileged” TME 12. Moreover, interactions between the tumor cells and the surrounding stroma create an inflammatory TME that is conducive to tumor growth and progression 13, 14. To successfully manipulate the immune system for effective PDA treatment, it is necessary to investigate the status of endogenous immune responses in the developing tumor.

Tumor cells also have diverse ways to escape from host immune surveillance. The expression of PD‐L1 and the downregulation of human leukocyte antigen (HLA) class I by tumor cells are crucial factors for the tumor development process 15, although complete loss of HLA‐class I triggers an natural killer (NK) cell response, which is directed against cells not expressing HLA‐class I (the “missing self” response) 16. PD‐L1 expression by tumor cells suppresses the proliferative and effector responses of T cells by engaging the inhibitory PD‐1 receptor expressed by activated T cells. Furthermore, expression of HLA class I molecules on tumor cell surfaces is critical for tumor‐specific T cells to recognize and attack the tumor. In addition, the antigen‐specific tumor immune response can also be modulated by the de novo expression of HLA class II antigens by tumor cells, which has been reported in a number of malignancies 17. The primary function of HLA class II is to present antigens to CD4^+^ T cells; however, this interaction can be stimulatory or inhibitory, and may lead to the induction of anergic CD4^+^ T cells, the activation of T_reg_, or the suppression of activated T cells by interaction with lymphocyte activating 18, 19, 20. To the best of our knowledge, there is no report showing a correlation between the expression of HLA class I and class II, PD‐L1, and the inflammatory conditions in PDA.

In this study, we characterized PDA infiltrating hematopoietic cells and determined the expression of PD‐L1 and HLA by PDA tumor cells to determine the immunological status of the PDA TME, its immune escape systems, and the impact of these factors on the patients’ prognosis.

## Materials and Methods

### Patients

Tumor samples were obtained from 36 patients (mean age 68.2 years) who had undergone pancreatic resection for PDA at the Department of Surgery and Science, Kyushu University Hospital, between February 1998 and December 2013. Tumors were diagnosed histologically based on the General Rules for the Study of Pancreatic Cancer by Japan Pancreas society (2009). All patients provided full written informed consent, and the study was approved by the Ethics Committee of Kyushu university (ID number: 27‐48). The baseline characteristics of patients were shown in Table 1.

**Table 1:** Baseline characteristics of PDA patients who underwent pancreatic resection

Factors	
Sex, male (%)	23 (63.9)
Age (years)	68.2 (51–89)
CEA (ng/ml)	5.5 (0.5–45.7)
CA19‐9 (U/ml)	477 (0.6–5449)
Tumor size (cm)	3.1 (1.5–6.5)
pT4 (%)	11 (30.5)
pN1 (%)	29 (80.6)
UICC staging ≥III (%)	12 (33.3)
Histologic grade Grade ≥2 (%)	11 (30.5)
Lymphatic invasion (%)	21 (58.3)
Vascular invasion (%)	8 (22.2)
Perineural invasion (%)	5 (13.9)

HLA, human leukocyte antingen; CEA, carcinoembryonic antigen; CA19‐9, carbohydrate antigen 19‐9; UICC, Union for International Cancer Control.

### Immunohistochemistry

Formalin‐fixed, paraffin‐embedded tumor sections were assessed immunohistochemically using monoclonal antibodies against HLA class I (EMR8‐5, 1:500; MBL, Tokyo, Japan), HLA DR (ab92511, 1:250; Abcam, Cambridge, England), PD‐L1 (SP142, 1:100; Spring Bioscience, CA), PD‐1 (ab137132, 1:250; Abcam), CD4 (4B12, 1:50; Dako, Glostrup, Denmark), CD8 (C8/144B, 1:50; Dako), CD56 (123C3, 1:50; Dako), CD68 (KP‐1, 1:300; Dako), and FoxP3 (ab20034, 1:100; Abcam), and the streptavidin–biotin‐peroxidase complex method. The staining with those monoclonal antibodies is described in Data S1. Staining intensity and percentage of stained cells were evaluated by two investigators, including one general pathologist, who was blinded to any information on the samples. The method used to determine the immunoreactivity score for HLA class I expression on the membrane of cancer cells was described previously 21. The expression of HLA‐DR was defined as positive when the intensity of the cell membrane and cytoplasm was higher than non‐neoplastic pancreatic ductal cells. The expression of PD‐L1 on the membrane and cytoplasm of cancer cells was defined as positive when the percentage of positive cells was ≥5% of PDA cells. The expression of PD‐1 was evaluated using a semi‐quantitative 0–5 scoring system (0 = negative, 1 = rare, 2 = low, 3 = moderate, 4 = high, 5 = very high), and the score ≥4 was defined as high PD‐1 expression as previously reported 22. Results from staining with antibodies specific for CD4, CD8, CD56, CD68, and FoxP3 were calculated by counting the number of stained infiltrating cells in three 40 ×  high‐power fields in the hot spot areas within non‐necrotic tumor tissues. Lymphocytes, as identified by their relative small size compared with macrophages and dendritic cells in histological section, were assessed for expression of CD4, CD8, and CD56.

### Statistical analysis

All statistical analyses were performed using SAS software (JMP 11.0.1; SAS Institute Inc., Cary, NC). Continuous variables were expressed as means ± standard deviations and compared using Mann–Whitney *U*‐tests. Categorical variables were compared using chi‐square tests. Overall and recurrence‐free survival rates were calculated with the Kaplan–Meier method, with between‐group differences compared using the log‐rank test. In risk factor analysis, propensity score matching analysis were performed to reduce the confounding. After balancing the two groups based on propensity scores which were calculated with Cox regression analysis for age, sex, and UICC stage, the risk factors of patient survival were evaluated using the Cox proportional hazard model. A *P *< 0.05 was considered statistically significant.

## Results

### Immune cells infiltrating primary PDA lesions

**Figure 1:**
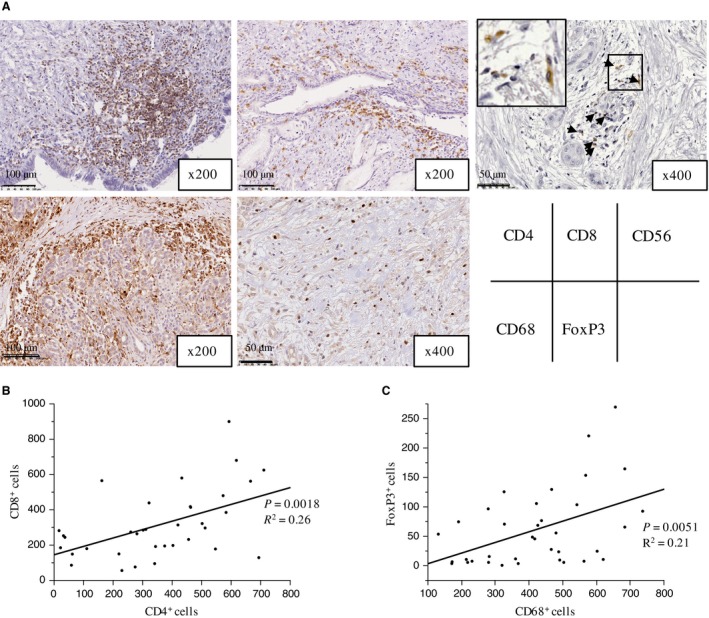
Immune cell infiltrates in primary PDA lesions. (A) Representative images for CD4, CD8, CD56, CD68 and FoxP3 immunohistochemistry in primary PDA lesions. (B) Association between the number of tumor‐infiltrating CD4^+^ cells and CD8^+^ cells. (C) Association between the number of tumor‐infiltrating CD68^+^ cells and FoxP3^+^ cells. The number of positive cells shown is the total number of positive cells within three high‐power fields.

The infiltration of PDA tumors by lymphocytes and macrophages was analyzed and their expression of CD4^+^, CD8^+^, CD56^+^, CD68^+^, and FoxP3^+^ cells was determined in histological sections (Fig. 1A). We confirmed that FoxP3^+^ cells also expressed CD4 in serial sections of the same sample. There were positive correlations between the number of CD8^+^ lymphocytes and CD4^+^ lymphocytes (*r* ^*2*^ = 0.260, *P *=* *0.002, Fig. 1B) and between the number of CD68^+^ cells and FoxP3^+^ cells (*r* ^*2 *^= 0.210, *P *=* *0.005, Fig. 1C). No other significant correlations were found. Interestingly, microscopic findings showed co‐infiltration of CD4^+^ and CD8^+^ lymphocytes in the tumor area in 27 of 36 cases, and CD68^+^ cells and FoxP3^+^ cells were diffusely infiltrated in the tumors (Fig. S1).

### The association between HLA class I or HLA‐DR expression and lymphocyte infiltration in primary PDA lesions

**Figure 2:**
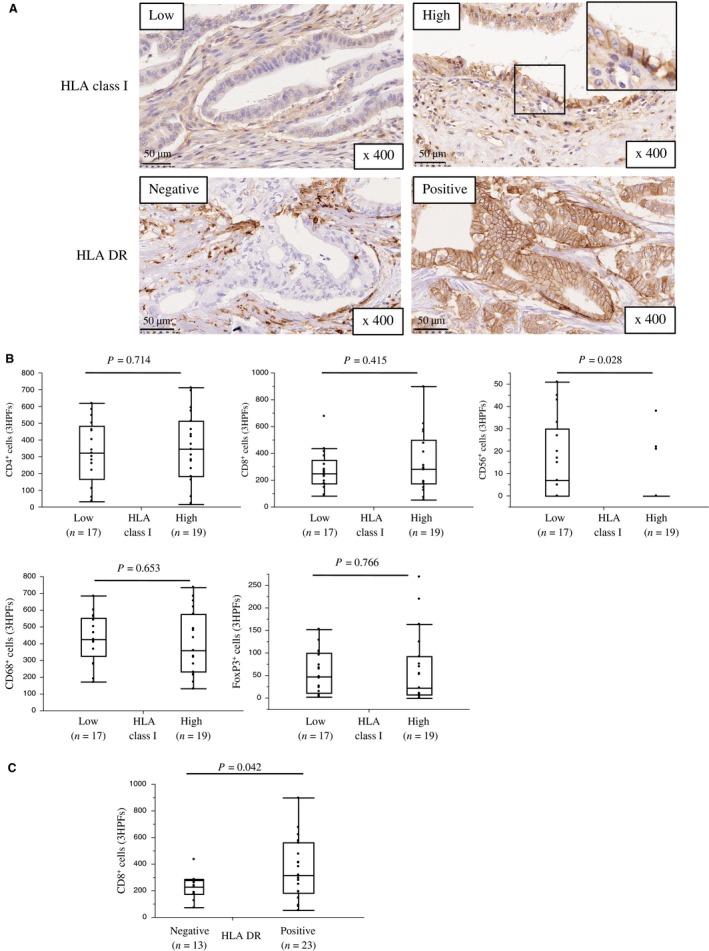
HLA expression and the association between HLA expression and immune cell infiltrates in primary PDA lesions. (A) Representative staining patterns for HLA class I and HLA‐DR immunohistochemistry in primary PDA lesions. (B) Association between HLA class I expression and the number of tumor‐infiltrating cells. (C) Association between HLA‐DR expression and the number of tumor‐infiltrating CD8^+^ cells. On each box, the central mark is the median, and the edges of the box are the 25th and 75th percentiles. Dots represent individual patients. The number of positive cells shown is the total number of positive cells in three high‐power fields.

We assessed the membrane expression of HLA class I on PDA cells to determine if HLA class 1 downregulation is a method of escape from CD8^+^ T‐cell immune responses. Among 36 cases, 19 (52.7%) were high, and 17 (47.3%) were low for HLA class I expression (Fig. 2A). HLA class I expression was inversely associated with CD56^+^ lymphocyte infiltrates (HLA class I high: mean *n* = 4 ± 3 vs. HLA class I low: mean *n* = 15 ± 3, *P *=* *0.028) (Fig. 2B). There was no significant correlation between HLA class I expression and the number infiltrating CD4^+^ lymphocytes (HLA class I low: mean *n* = 327 ± 49 vs. HLA class I high: mean *n* = 352 ± 46, *P *=* *0.712) or CD8^+^ lymphocytes (HLA class I low: mean *n* = 273 ± 46 vs. HLA class I high: mean *n* = 326 ± 45, *P *=* *0.415). Although HLA‐DR was not expressed on normal pancreatic ductal cells (Fig. S2), positive expression of HLA‐DR by PDA cells was identified in 23 cases (63.9%; Fig. 2A). The number of infiltrating CD8^+^ lymphocytes were significantly higher in HLA‐DR positive PDA tissues than in HLA‐DR negative PDA tissues (HLA‐DR negative: mean *n* = 226 ± 51 vs. HLA‐DR positive: mean *n* = 361 ± 39, *P *=* *0.042; Fig. 2C).

### The expression of PD‐L1 on PDA cells, the presence of PD‐1 positive cells in PDA lesions, and the correlation with tumor‐infiltrating lymphocytes

**Figure 3:**
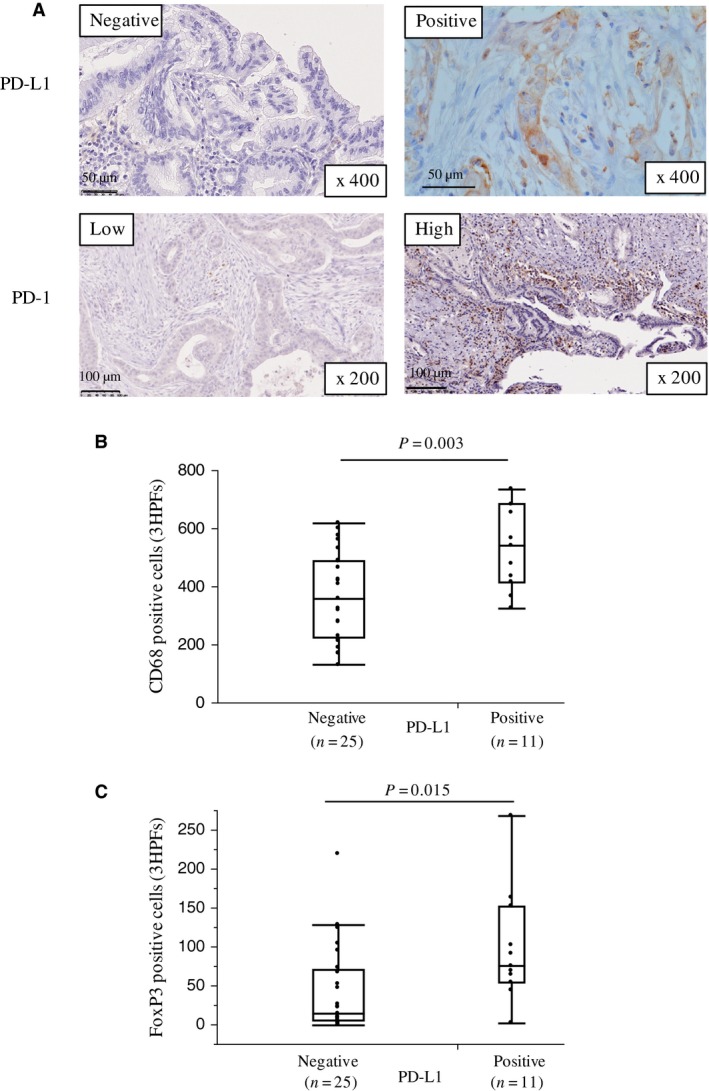
PD‐1 and PD‐L1 expression and the association between PD‐L1 expression and immune cell infiltrates in primary PDA lesions. (A) Representative staining patterns for PD‐1 and PD‐L1 immunohistochemistry in primary PDA lesions. (B, C) Association between PD‐L1 expression and the number of tumor‐infiltrating CD68^+^ cells (B) or FoxP3^+^ cells (C). On each box, the central mark is the median, and the edges of the box are the 25th and 75th percentiles. Dots represent individual patients. The number of positive cells shown is the total number of positive cells in three high‐power fields.

Immunohistochemical evaluation showed that PD‐L1 was expressed in the cytoplasm and on the cell membrane of PDA specimens (Fig. 3A). Among 36 cases, 11 (30.6%) PDA samples were positive for PD‐L1 and 25 (69.4%) were negative. PD‐L1 was also expressed on noncancerous cells, including tumor‐infiltrating lymphocytes. Although enhanced PD‐L1 expression on cancer cells was seen in the hot spot area of CD4^+^ or CD8^+^ lymphocyte infiltration in 6 of 11 (54.3%) PD‐L1 positive samples (Fig. S1), there was no significant correlation between PD‐L1 expression by PDA cells and the number of infiltrating CD4^+^ or CD8^+^ cells. However, there was a significant positive correlation between PD‐L1 expression by PDA cells and the number of infiltrating CD68^+^ (PD‐L1 negative: mean *n* = 369 ± 29 vs. PD‐L1 positive: mean *n* = 537 ± 44, *P *=* *0.003; Fig. 3B) and FoxP3^+^ cells (PD‐L1 negative: mean *n* = 43 ± 12 vs. PD‐L1 positive: mean *n* = 99 ± 18, *P *=* *0.015; Fig. 3C). Enhanced PD‐L1 expression on PDA cells was also observed in the areas where CD68^+^ cells, and neither CD4^+^ nor CD8^+^ lymphocytes were infiltrating in the tumors (Fig. S3). The areas of CD68^+^ cell infiltration were more abundant than those of CD4^+^ or CD8^+^ lymphocytes. On the other hands, FoxP3^+^ cells diffusely infiltrated the PDA lesion (Fig. 1A and S1).

PD‐1 was expressed on tumor‐infiltrating lymphocytes in all tumors but not on PDA cells (Fig. 3A). High PD‐1 expression was detected in 16 (44.4%) samples. The prevalence of PD‐1^+^ cells correlated with the degree of lymphocyte infiltration. As expected, a statistically significant positive trend was found between PD‐1 expression and the number of infiltrating CD4^+^ (PD‐1 low: mean *n* = 296 ± 42 vs. PD‐1 high: mean *n* = 430 ± 47, *P *=* *0.042), CD8^+^ (PD‐1 low: mean *n* = 230 ± 40 vs. PD‐1 high: mean *n* = 408 ± 43, *P *=* *0.005), CD68^+^ (PD‐1 low: mean *n* = 371 ± 35 vs PD‐1 high: mean *n* = 482 ± 39, *P *=* *0.040), and FoxP3^+^ (PD‐1 low: mean *n* = 38 ± 13 vs. PD‐1 high: mean *n* = 88 ± 15, *P *=* *0.020) cells, but not CD56^+^ cells (PD‐1 low: mean *n* = 8 ± 3 vs. PD‐1 high: mean *n* = 12 ± 4, *P *=* *0.481) (Fig. S4).

### The correlation between HLA class I and PD‐L1 expression, the clinicopathological findings, and the impact on PDA patient prognosis

**Figure 4:**
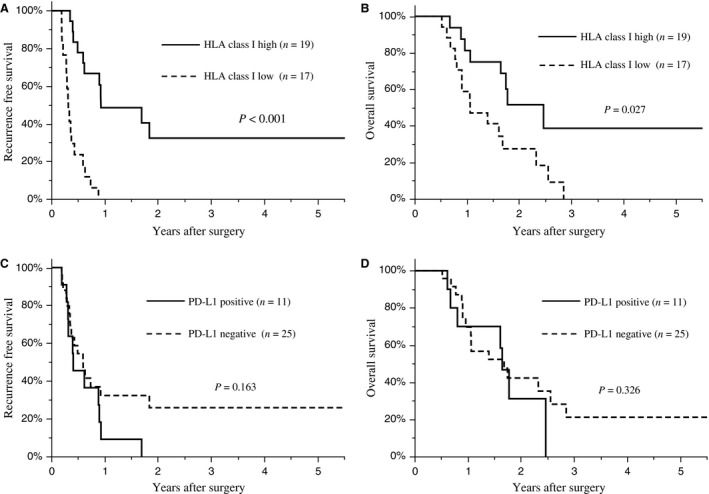
HLA class I or PD‐L1 expression and patient survival. Recurrence‐free survival rates (A) or overall survival rates (B) in PDA patients with high (solid line) or low (dotted line) HLA class I expressing tumors. Recurrence‐free survival rates (C) or overall survival rates (D) in PDA patients with positive (solid line) or negative (dotted line) PD‐L1 expression.

**Figure 5:**
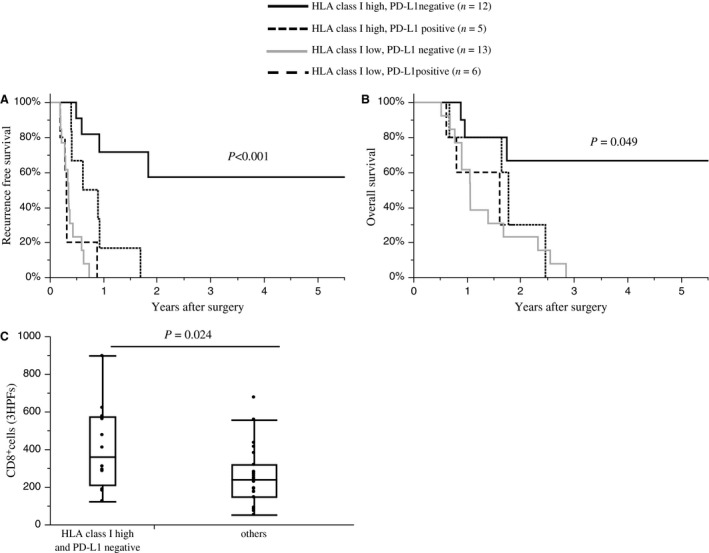
The expression patterns of HLA class I and PD‐L1 and the survival outcomes of PDA patients. Recurrence‐free survival rates (A) or overall survival rates (B) in PDA patients sorted by of HLA class I and PD‐L1 expression: HLA class I high/PD‐L1 negative, *n* = 12 (solid lines); HLA class I high/PD‐L1 positive, *n* = 5 (dotted lines); HLA class I low/PD‐L1 negative, *n* = 13 (gray line); HLA class I low/PD‐L1 positive, *n* = 6 (dashed line). (C) The number of tumor‐infiltrating CD8^+^ lymphocytes in PDA patients divided into two groups: HLA class I high/PD‐L1 negative, *n* = 12; and the remaining patients, *n* = 24. The number of positive cells shown is the total number of positive cells in three high‐power fields.

Finally, we investigated the correlation between HLA and PD‐L1 expression in PDAs, the clinicopathological findings, and the impact on patient survival. Tumors with pT4 (*P *=* *0.006) and with a more advanced clinical stage (*P *=* *0.018) were more frequently observed in HLA class I low PDA samples than in HLA class I high samples (Table S1). The risk factor analysis for poor survival, after balancing the cohorts using propensity score matching for age, sex, and UICC stage (Table S2), showed that low HLA class I expression was an only risk factor of poor recurrence‐free survival (Hazard ratio 6.10, *P *=* *0.001). PD‐L1 positive tumors were significantly associated with a higher histological grade than PD‐L1 negative tumors (*P *=* *0.032; Table S3). A survival analysis indicated that patients with HLA class I high tumors had better recurrence‐free survival (*P *<* *0.001; Fig. 4A) and overall survival (*P *=* *0.027; Fig. 4B) than those with HLA class I low tumors. HLA‐DR (Fig. S5) and PD‐L1 (Fig. 4C and D) expression by PDA cells, and PD‐1 expression (Fig. S5) in the tumor microenvironment had no association with survival. When patients were divided into four groups according to PD‐L1 and HLA class I expression, negative PD‐L1 expression and high HLA class I expression by PDA (*n* = 12) was associated with a significant better recurrence‐free survival (*P *<* *0.001; Fig. 5A) and overall survival (*P *=* *0.049; Fig. 5B) compared with patients HLA class I high and PD‐L1 positive (*n* = 5), HLA class I low and PD‐L1 negative (*n* = 13), or HLA class I low and PD‐L1 positive (*n* = 6) tumors. In this analysis, we also investigated infiltration of CD8 lymphocytes between the groups. The number of infiltrating CD8^+^ lymphocytes were significantly higher in tumors that were PD‐L1 negative and HLA class I high than in tumors with different PD‐L1 and HLA class I expression (PD‐L1 negative and HLA class I high: mean *n* = 412 ± 52 vs. different PD‐L1 and HLA class I expression: mean *n* = 259 ± 38, *P *=* *0.024; Fig. 5C). These findings suggest that PD‐L1 expression affects the prognosis of patients with HLA‐class I positive PDAs, and it is associated with a reduced infiltration of CD8^+^ lymphocytes.

### The association between membranous PD‐L1 expression and patients’ characters, tumor‐infiltrative lymphocytes, and prognosis

It is also well accepted that PD‐L1 positivity is assessed according to the membranous expression of PD‐L1. Therefore, we also evaluated the membranous expression of PD‐L1 on PDA and analyzed its correlation with other clinicopathological features. The membranous expression of PD‐L1 was observed in six cases (16.7%) of PDAs. Membranous PD‐L1‐positive tumors were significantly associated with a higher histological grade than negative tumors (*P *=* *0.035; Table S4). There were significant positive correlations between membranous PD‐L1 expression on PDA cells and the number of infiltrating CD68^+^ (membranous PD‐L1 negative: mean *n* = 392 ± 28 vs. membranous PD‐L1 positive: mean *n* = 563 ± 63, *P *=* *0.018; Fig. S6A) and FoxP3^+^ cells (membranous PD‐L1 negative: mean *n* = 48 ± 11 vs. membranous PD‐L1 positive: mean *n* = 121 ± 24, *P *=* *0.011; Fig. S6B). There is no significant difference between membranous PD‐L1 expressions and recurrence‐free survival (*P *=* *0.484; Fig. S7A) and overall survival (*P *=* *0.584; Fig. S7B). When patients were divided into four groups according to membranous PD‐L1 and HLA class I expression, negative membranous PD‐L1 expression and high HLA class I expression by PDA (*n* = 14) was associated with a significant better recurrence‐free survival (*P *<* *0.001; Fig. S8A) and overall survival (*p *=* *0.013; Fig. S8B) compared with patients HLA class I high and PD‐L1 positive (*n* = 4), HLA class I low and PD‐L1 negative (*n* = 16), or HLA class I low and PD‐L1 positive (*n* = 2) tumors. The number of infiltrating CD8^+^ lymphocytes were significantly higher in tumors that were membranous PD‐L1 negative and HLA class I high than in tumors with different PD‐L1 and HLA class I expression (membranous PD‐L1 negative and HLA class I high: mean *n* = 395 ± 49 vs. different membranous PD‐L1 and HLA class I expression: mean *n* = 245 ± 37, *P *=* *0.021; Fig. S8C).

## Discussion

PDA is associated with an immunosuppressive TME that promotes the growth and progression of tumors 7, 8, 9, 10, 23. Tumors have many immune escape systems, including by expressing PD‐L1 and downregulating HLA class I 17, 21, 22. We demonstrated that high expression of HLA class I by PDA cells is a good prognostic factor for PDA patients, and that PD‐L1 expression affects the prognosis of patients with HLA‐class I positive PDAs.

Our histological evaluation of inflammatory cells suggests TME of PDA consists of heterogeneous cell populations and immunostimulatory and immunosuppressive condition may be admixed. In a genetically engineered mouse PDA model 10, accumulation of macrophages, myeloid‐derived suppressor cells, and T_reg_ was detected with disease progression in TME of spontaneous tumor. In addition, co‐infiltration of CD4^+^ and CD8^+^ T cells was also detected with disease progression and the infiltration of both CD4^+^ and CD8^+^ T cells were negatively correlated with macrophage infiltration with a mutually exclusion in TME in the mouse model 10. In line with the findings, correlation between CD4^+^ and CD8^+^ T‐cell infiltration was detected in PDA patients, but not that between CD8^+^ T‐cell infiltration and Foxp3^+^ T_reg_, suggesting the effector CD4^+^ T cell may infiltrate into the TME of PDA associated with CD8^+^ T‐cell infiltration, irrespective of T_reg_ infiltration. In addition, positive association between the number of infiltrated CD68^+^ cells and FoxP3^+^ cells, but not between CD68^+^ cells and CD4‐ and CD8‐T cell‐infiltration, was also detected in PDA patients. These findings suggest there are two TME in PDA, namely immunostimulatory and immunosuppressive TME. The predominant TME might affect the prognosis of patients.

Some reports have previously shown an association between PD‐L1 expression by tumors and immune cell infiltration, particularly in melanoma tumors 24, and the increased expression of PD‐L1 was dependent on CD8^+^ T cells and interferon‐*γ* 25. Type II interferons, a signature of T‐cell activation, not only leads to the expression of PD‐1 and PD‐L1, but also to other immunosuppressive factors, such as indoleamine‐pyrrole 2,3‐dioxygenase production and the active presence of T_reg_ 26. The negative feedback generated by these inhibitory pathways is an adaptive resistance process that follows T‐cell infiltration. There have been two controversial reports on PD‐L1 expression by PDA cells: Nomi et al. reported that PD‐L1 expression by PDA inversely correlates with CD8^+^ T‐cell infiltration into the TME 27, whereas Zhu et al. reported that blocking colony‐stimulating factor 1 or its receptor increased the number of infiltrating CD4^+^ and CD8^+^ T cells, which induced PD‐L1 expression by the PDA cells 28. Our study suggests that both the studies may be true. Indeed, of all 11 PD‐L1 positive samples, six (54.5%) had a strong association between PD‐L1 expression by the PDA and the number of CD4^+^ and/or CD8^+^ lymphocytes infiltrating the TME. However, because the foci of infiltration of CD4^+^ or CD8^+^ lymphocytes were relatively small compared with the foci for CD68^+^ cells, we did not obtain a statistically significant correlation between PD‐L1 expression and T‐cell infiltration. PD‐L1 expression by PDA cells was significantly correlated with infiltration by immunosuppressive cells, such as macrophages and T_reg_. We have previously reported similar findings in hepatocellular carcinoma, in which PD‐L1 expression was correlated with infiltration of CD163^+^ cells 21. According to our previous reports, the tumor‐infiltrated macrophages expressed IL‐6 and HCC cells in the same area exhibited activation of signal transducer and activator of transcription (STAT) 3 29. Therefore, it is possible that the IFN‐*γ*‐STAT1 axis induced by CD4^+^ or CD8^+^ T cells, or macrophage‐producing IL‐6‐STAT3 axis, might serve as the primary mechanism of PD‐L1 expression by PDA cells and T_regs_ infiltration into the TME 29, 30. In fact, both IFN‐*γ* and IL‐6 induce PD‐L1 expression in many cancer cell lines 31. Therefore, there could be two possible mechanisms for PD‐L1 expression, and this study suggests that the latter mechanism is predominant in the TME of PDA, at least under untreated conditions.

This study showed that HLA class I expression was statistically correlated with the prognosis of PDA patients. This finding is not unique to PDA, as we and others have reached the same conclusion in patients with hepatocellular carcinoma and intrahepatic cholangiocellular carcinoma 21, 22. These results suggest that the expression pattern of these molecules by tumor cells could determine the immunological conditions of the TME favorable for patient survival. In addition, we indicated that PD‐L1 negative and HLA class I high expressing PDA was infiltrated by more CD8^+^ T cell and was associated with a better prognosis than PDA with different PD‐L1 and HLA class I expression. PD‐L1 drives CD8^+^ T cells into an exhausted state and can also induce their apoptosis 12. High HLA class I expression by intrahepatic cholangiocellular carcinoma was previously reported to be positively associated with CD8^+^ T‐cell infiltration 22. In line with the opposite PD‐L1 expression mechanism mentioned above, infiltration of both lymphocytes and macrophages were associated with higher expression of PD‐L1. Therefore, our results suggest that, especially in HLA class I‐positive PDA, the immunosuppressive and immunostimulatory balance in the TME is important, and the predominant environment might define the immunological status of PDA that affects patient prognosis.

There have been quite a few published studies describing the expression of PD‐L1 expression in human PDA (Table 2). The cytoplasmic and membranous expression rate of PD‐L1 in PDAs was reported to be 39.2–63.3% 23, 27, 32 and membranous expression of PD‐L1 were 28.7% 33, the percentages were higher than this report. These differences might be attributable to the use of different antibodies and interpretation criteria in the lack of consensus PD‐L1 immunohistochemistry method at present.

**Table 2:** Recent reports regarding PD‐L1 expression by immunohistochemical analysis in human PDA

Ref	Author	Year	*n*	Antibody clone	Locus	Positive criteria	Positive rate (%)
27	Nomi et al.	2007	51	MIH1	Cytoplasmic, membranous	≥10% in the total tumor area	39.2
32	Wang et al.	2010	81	MIH1	Cytoplasmic, membranous	>5% in the total tumor area	49.4
23	Hutcheson et al.	2016	158	E1L3N	Cytoplasmic, membranous	≥10% in the total tumor area	63.3
33	Wang et al.	2017	94	No data	Membranous	Comprehensive score	28.7
	This study	2017	36	SP142	Cytoplasmic, membranous	>5% in the total tumor area	30.6
	This study	2017	36	SP142	Membranous	>5% in the total tumor area	16.7

Downregulation of HLA class I enables tumors to escape immune surveillance 21, 34, although tumor cells not expressing HLA class I are susceptible to attack by NK cells because HLA class I is an inhibitory signal for NK cells that prevents NK cell‐mediated lysis 16. This phenomenon may explain the inverse correlation between HLA class I expression and CD56^+^ cell infiltration in our study.

PD‐1 is expressed on a large proportion of tumor‐infiltrating immune cells, such as T cells, T_regs_, B cells, activated monocytes, dendritic cells, NK cells, and natural killer T cells 35, 36, 37. It is convincing that the number of infiltrated immune cells, excluding CD56^+^ lymphocytes, correlated with PD‐1 expression, suggesting that infiltrating lymphocytes are exhausted. Because the number of infiltrating CD56^+^ lymphocytes were relatively low compared with other inflammatory cells in PDA, the correlation between PD‐1 expression and the number of infiltrating CD56^+^ lymphocytes may not have been detected.

Although a few types of non‐neoplastic cells, including B lymphocytes, antigen‐presenting cells and activated T lymphocytes, constitutively express HLA class II antigens, some epithelial cells can also express HLA‐DR under certain circumstances, such as infection, autoimmune disease, or benign or malignant transformation 38. The de novo expression of HLA class II by cancer cells is also affected by cytokines produced by inflammatory cells, such as IFN‐*γ* 39, 40, 41. HLA class II expression by PDA cells has been studied by Scupoli et al., in which de novo HLA class II expression was identified in only 3 of 8 cases (37.5%) 41. They reported a hierarchy in the expression of HLA class II (HLA‐DR>HLA‐DP>HLA‐DQ). HLA‐DQ was never expressed in cancer tissue, and HLA‐DR composed a large part of the HLA class II expression in PDA. This trend was also observed in other cancer types 39, 40. In this study, although we could also detect HLA‐DR expression in 23 of 36 PDA cases, the expression of HLA‐DR was heterogeneous in HLA‐DR positive PDA. Therefore, de novo HLA‐DR expression may be affected by the TME, including T‐cell responses. The positive association between HLA‐DR expression and CD8^+^ T‐cell infiltration suggests that HLA class II expression by PDA cells may be enhanced by the infiltration of CD8^+^ lymphocytes. Unfortunately, we could not observe the prognostic impact of HLA‐DR expression by PDA cells in this study.

Various strategies, such as cell‐based cancer vaccines (GVAX), CD40 agonistic antibodies, and colony‐stimulating factor 1 receptor blockade, have been developed to transform the immunosuppressive pancreatic TME into conditions that can empower an anticancer immune response 28, 42, 43. These strategies were able to induce the formation of tertiary lymphoid aggregates in patients with PDA, resulting in the upregulation of the PD‐1/PD‐L1 pathway. This condition could be favorable for blocking immune checkpoints to induce stronger immune responses in the tumor environment. At present, there is no good biomarker to predict the effect of anti‐PD‐1/PD‐L1 blockade. Recent clinical trials assessing the efficacy and safety of anti‐PD‐1/PD‐L1 antibody blockade suggest that higher PD‐L1 expression by patient cancer cells was associated with higher objective responses 3, 44. However, some patients with PD‐L1 negative cancer also had significant objective responses after treatment with the antibodies 3, 44. HLA class I expression on cancer cells is important to be recognized by tumor associate antigen‐recognizing CD8^+^ T cells. Here, our data indicated the stratified group of patients with highly HLA‐class I‐expressed PDA showed significant difference in prognosis between PD‐L1‐positive PDA and PD‐L1‐negative one, but not the stratified group of patients with low class I‐expressed PDA. In other words, the status of PD‐L1 expression affect prognosis only in patients with highly HLA‐class I‐expressed cancers, but not in those with low HLA‐class I‐expressed cancers. Since Anti‐PD‐1/PD‐L1 treatment is targeting the exhausted CD8^+^ T cells and turn the exhausted CD8^+^ T cells into active state, which is dependent on CD28 45, 46, injection to patients with anti‐PD‐1/PD‐L1 antibodies is presumed to make the status of PD‐L1‐positive cancerous mass to the PD‐L1‐negative one, resulting in that infiltrating CD8 T cells would attack HLA‐class I‐positive PDA and improve patient prognosis. We obtained similar findings in other cancer 21, 47. Therefore, we have consistently hypothesized that expression of HLA class I antigen theoretically has a potential role as a biomarker for patients who are suitable for anti‐PD‐1/PD‐L1 antibody treatment.

In conclusion, the TME of PDA may enable the evasion of the immune system by upregulating PD‐L1. Evaluation of both PD‐L1 and HLA class I expression on PDA cells may be a good predictor of prognosis for PDA patients. HLA class I expression by tumor cells should be evaluated when selecting PDA patients who may be eligible for treatment with PD‐1/PD‐L1 immune checkpoint blockade therapies.

## Conflict of Interest

The authors declare no potential conflicts of interest.

## Supporting information


**Figure S1.** Enhanced PD‐L1 expression by PDA cells in areas of CD4^+^ or CD8^+^ T‐cell infiltration.

**Figure S2.** HLA‐DR expression in normal pancreatic ductal cells.

**Figure S3.** Enhanced PD‐L1 expression by PDA cells in areas of CD68^+^ cell infiltration.

**Figure S4.** The association between PD‐1 expression and immune cell infiltrates in primary PDA lesions

**Figure S5.** HLA‐DR or PD‐1 expression and patient survival

**Figure S6.** The association between membranous PD‐L1 expression and immune cell infiltrates in primary PDA lesions.

**Figure S7.** Membranous PD‐L1 expression and patient survival.

**Figure S8.** The expression patterns of HLA class I and *membranous* PD‐L1 and the survival outcomes of PDA patients.

**Table S1.** Baseline characteristics of PDA patients with high or low HLA class I expression who underwent pancreatic resection.

**Table S2.** Baseline characteristics of PDA patients with high or low HLA class I expression who underwent pancreatic resection, after matching.

**Table S3.** Baseline characteristics of PDA patients with negative or positive PD‐L1 expression who underwent pancreatic resection.

**Table S4.** Baseline characteristics of PDA patients with negative or positive *membranous* PD‐L1 expression who underwent pancreatic resection.

**Data S1.** Immunohistochemical staining.
