# Supplementary material for: The prognostic impact of programmed cell death ligand 1 and human leukocyte antigen class I in pancreatic cancer
Source: Cancer Med. 2017 Jun 10;6(7):1614–26. doi: 10.1002/cam4.1087 (PMC5504334; doi:10.1002/cam4.1087)
Supplement: Supplementary file 1 — Figure S1. Enhanced PD‐L1 expression by PDA cells in areas of CD4+ or CD8+ T‐cell infiltration. [file CAM4-6-1614-s001.docx]

**Figure S1. Enhanced PD-L1 expression by PDA cells in areas of CD4^+^ or CD8^+^ T cell infiltration**


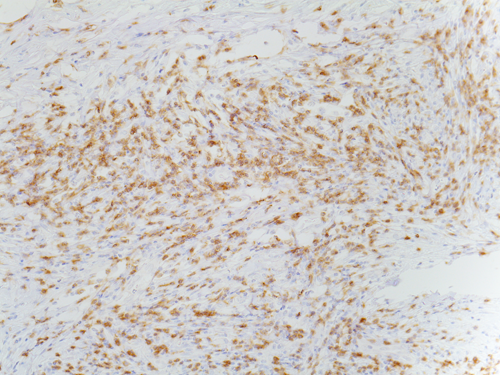

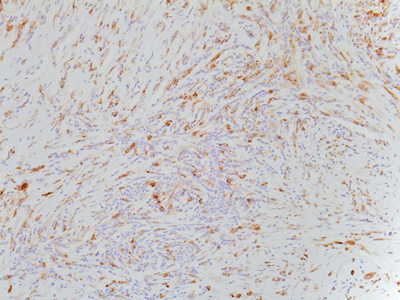

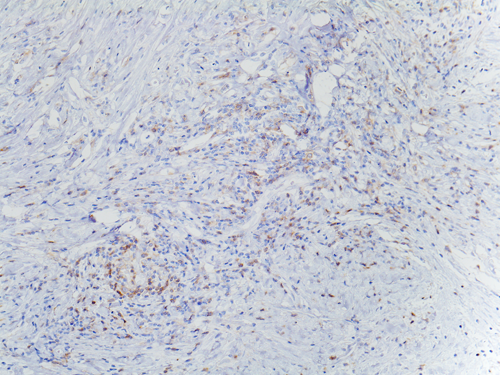

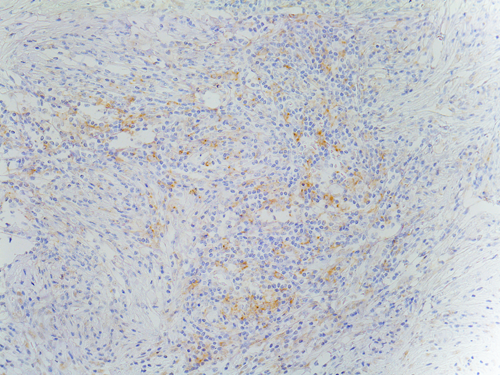

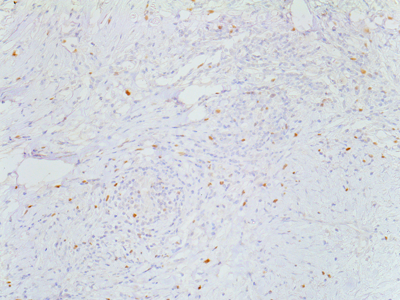


×200

×200

×200

CD4

CD8

× 200

PD-L1

CD68

FoxP3

×200

Representative immunohistochemistry staining patterns of CD4, CD8, CD68, FoxP3 and PD-L1 in serial sections of the same primary PDA lesion.
