# Supplementary material for: The prognostic impact of programmed cell death ligand 1 and human leukocyte antigen class I in pancreatic cancer
Source: Cancer Med. 2017 Jun 10;6(7):1614–26. doi: 10.1002/cam4.1087 (PMC5504334; doi:10.1002/cam4.1087)
Supplement: Supplementary file 2 — Figure S2. HLA‐DR expression in normal pancreatic ductal cells. [file CAM4-6-1614-s002.docx]

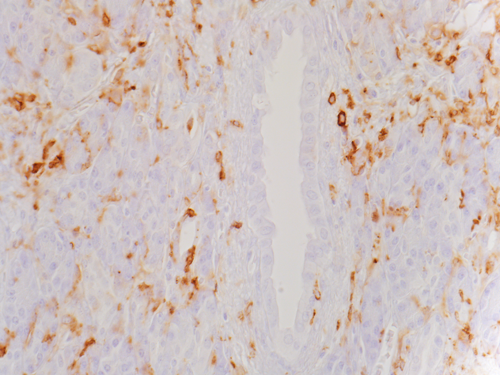


×400

**Figure S2. HLA-DR expression in normal pancreatic ductal cells**

A representative immunohistochemistry image of a normal pancreatic duct (arrows) with HLA-DR staining.
