# Supplementary material for: The prognostic impact of programmed cell death ligand 1 and human leukocyte antigen class I in pancreatic cancer
Source: Cancer Med. 2017 Jun 10;6(7):1614–26. doi: 10.1002/cam4.1087 (PMC5504334; doi:10.1002/cam4.1087)
Supplement: Supplementary file 3 — Figure S3. Enhanced PD‐L1 expression by PDA cells in areas of CD68+ cell infiltration. [file CAM4-6-1614-s003.docx]

**Figure S3. Enhanced PD-L1 expression by PDA cells in areas of CD68^+^ cell infiltration**


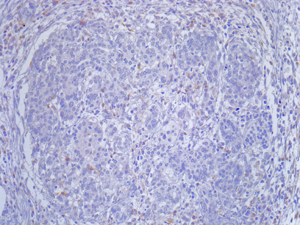

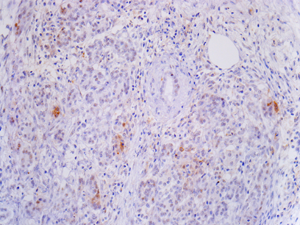

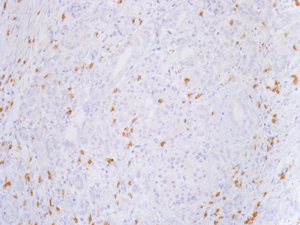

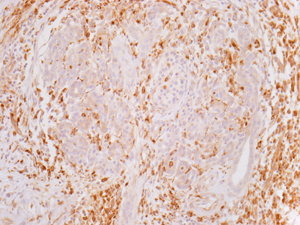


×200

×200

×200

CD4

CD8

×200

PD-L1

CD68

Representative immunohistochemistry staining patterns of CD4, CD8, CD68 and PD-L1 in serial sections of the same primary PDA lesion.
