# Supplementary material for: The prognostic impact of programmed cell death ligand 1 and human leukocyte antigen class I in pancreatic cancer
Source: Cancer Med. 2017 Jun 10;6(7):1614–26. doi: 10.1002/cam4.1087 (PMC5504334; doi:10.1002/cam4.1087)
Supplement: Supplementary file 5 — Figure S5. HLA‐DR or PD‐1 expression and patient survival [file CAM4-6-1614-s005.docx]

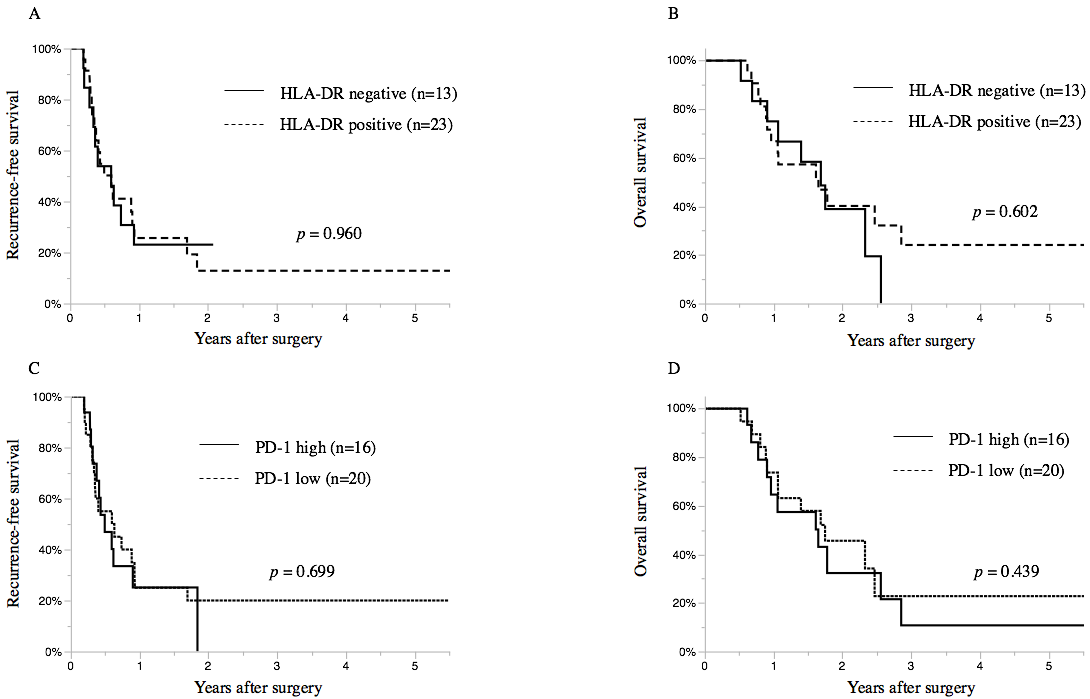
**Figure S5.** **HLA-DR or PD-1 expression and patient survival**

Recurrence-free survival rates (A) and overall survival rates (B) of PDA patients with negative (solid line) or positive (dotted line) HLA-DR expressing tumors. Recurrence-free survival rates (C) and overall survival rates (D) of PDA patients with high (solid line) or low (dotted line) PD-1 expressing tumors.
