# Supplementary material for: The prognostic impact of programmed cell death ligand 1 and human leukocyte antigen class I in pancreatic cancer
Source: Cancer Med. 2017 Jun 10;6(7):1614–26. doi: 10.1002/cam4.1087 (PMC5504334; doi:10.1002/cam4.1087)
Supplement: Supplementary file 6 — Figure S6. The association between membranous PD‐L1 expression and immune cell infiltrates in primary PDA lesions. [file CAM4-6-1614-s006.docx]

**
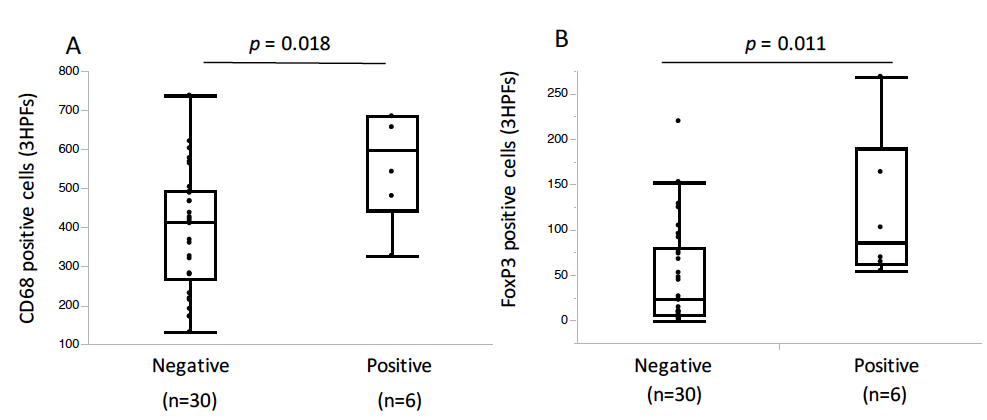
**

**Figure S6.** **The association between *membranous* PD-L1 expression and immune cell infiltrates in primary PDA lesions**

Association between *membranous* PD-L1 expression and the number of tumor-infiltrative CD68^+^ cells (A) and FoxP3^+^ cells (B). On each box, the central mark is the median, and the edges of the box are the 25th and 75th percentiles. Each dot represents an individual patient. The number of positive cells shown is the total number of positive cells in three high-power fields.
