# Supplementary material for: The prognostic impact of programmed cell death ligand 1 and human leukocyte antigen class I in pancreatic cancer
Source: Cancer Med. 2017 Jun 10;6(7):1614–26. doi: 10.1002/cam4.1087 (PMC5504334; doi:10.1002/cam4.1087)
Supplement: Supplementary file 7 — Figure S7. Membranous PD‐L1 expression and patient survival. [file CAM4-6-1614-s007.docx]

**
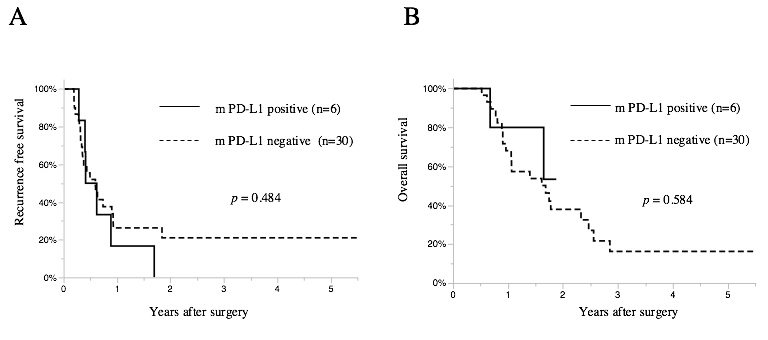
**

**Figure S7.** ***membranous* PD-L1 expression and patient survival**

Recurrence-free survival rates (A) and overall survival rates (B) of PDA patients with negative (solid line) or positive (dotted line) *membranous* PD-L1 expressing tumors.
