# Supplementary material for: The prognostic impact of programmed cell death ligand 1 and human leukocyte antigen class I in pancreatic cancer
Source: Cancer Med. 2017 Jun 10;6(7):1614–26. doi: 10.1002/cam4.1087 (PMC5504334; doi:10.1002/cam4.1087)
Supplement: Supplementary file 8 — Figure S8. The expression patterns of HLA class I and membranous PD‐L1 and the survival outcomes of PDA patients. [file CAM4-6-1614-s008.docx]

A

Years after surgery

Recurrence free survival

*p*<0.001

Years after surgery

Overall survival

*p*= 0.013

B

HLA class I high, mPD-L1 negative (n=14)

HLA class I high, mPD-L1 positive (n=4)

HLA class I low, mPD-L1 negative (n=16)

HLA class I low, mPD-L1 positive (n=2)

C

*p* = 0.021

others

(n = 22)

CD8^+^ cells (3HPFs)

HLA class I high

and mPD-L1 negative

(n = 14)

**Supplementary Figure 8.** **The expression patterns of** **HLA class I and *membranous* PD-L1 and the survival outcomes of PDA patients**

Recurrence-free survival rates (A) or overall survival rates (B) in PDA patients sorted by of HLA class I and *membranous* PD-L1 expression (mPD-L1): HLA class I high/mPD-L1 negative, n=14 (solid lines); HLA class I high/mPD-L1 positive, n=4 (dotted lines); HLA class I low/mPD-L1 negative, n=16 (grey line); HLA class I low/mPD-L1 positive, n=2 (dashed line). (C) The number of tumor-infiltrating CD8^+^ lymphocytes in PDA patients divided into two groups: HLA class I high/mPD-L1 negative, n=14; and the remaining patients, n=22. The number of positive cells shown is the total number of positive cells in three high-power fields.
