# Supplementary material for: The prognostic impact of programmed cell death ligand 1 and human leukocyte antigen class I in pancreatic cancer
Source: Cancer Med. 2017 Jun 10;6(7):1614–26. doi: 10.1002/cam4.1087 (PMC5504334; doi:10.1002/cam4.1087)
Supplement: Supplementary file 9 — Table S1. Baseline characteristics of PDA patients with high or low HLA class I expression who underwent pancreatic resection. [file CAM4-6-1614-s009.docx]

**Supplementary Table S1.** Baseline characteristics of PDA patients with high or low HLA class I expression who underwent pancreatic resection

| Factors | HLA class I | | *p*-value |
| --- | --- | --- | --- |
|  | High (n=19) | Low (n=17) |  |
| Gender, male, n (%) | 12 (63.2) | 11 (64.7) | 0.923 |
| Age (years) | 68 ± 2 | 69 ± 2 | 0.854 |
| CEA (ng/ml) | 3.2 ± 2.0 | 7.7 ± 2.0 | 0.124 |
| CA19-9 (U/ml) | 187 ± 285 | 734 ± 268 | 0.173 |
| Tumor size (cm) | 2.7 ± 0.3 | 3.5 ± 0.3 | 0.052 |
| pT4, n (%) | 2 (10.5) | 9 (52.9) | 0.006 |
| pN1, n (%) | 14 (73.7) | 15 (88.2) | 0.271 |
| UICC staging ≥III, n (%) | 3 (15.8) | 9 (52.9) | 0.018 |
| Histologic grade ≥2, n (%) | 7 (36.8) | 4 (23.5) | 0.481 |
| Lymphatic invasion, n (%) | 11 (57.9) | 10 (62.5) | 0.782 |
| Vascular invasion, n (%) | 2 (11.1) | 6 (37.5) | 0.070 |
| Perineural invasion, n (%) | 1 (5.3) | 4 (23.5) | 0.164 |

HLA, human leukocyte antigen; CEA, carcinoembryonic antigen; CA19-9, carbohydrate antigen 19-9; UICC, Union for International Cancer Control.
