# Supplementary material for: The prognostic impact of programmed cell death ligand 1 and human leukocyte antigen class I in pancreatic cancer
Source: Cancer Med. 2017 Jun 10;6(7):1614–26. doi: 10.1002/cam4.1087 (PMC5504334; doi:10.1002/cam4.1087)
Supplement: Supplementary file 10 — Table S2. Baseline characteristics of PDA patients with high or low HLA class I expression who underwent pancreatic resection, after matching. [file CAM4-6-1614-s010.docx]

**Supplementary Table S2.** Baseline characteristics of PDA patients with high or low HLA class I expression who underwent pancreatic resection, after matching

| Factors | HLA class I | | *p*-value |
| --- | --- | --- | --- |
|  | High (n=11) | Low (n=11) |  |
| Gender, male, n (%) | 6 (54.6) | 6 (54.6) | 1.000 |
| Age (years) | 68 ± 3 | 69 ± 3 | 0.772 |
| CEA (ng/ml) | 3.3 ± 3.4 | 9.1 ± 3.2 | 0.228 |
| CA19-9 (U/ml) | 154 ± 441 | 563 ± 376 | 0.491 |
| Tumor size (cm) | 2.9 ± 0.4 | 3.4 ± 0.4 | 0.331 |
| pT4, n (%) | 2 (18.2) | 3 (27.3) | 0.611 |
| pN1, n (%) | 8 (72.7) | 9 (81.8) | 0.611 |
| UICC staging ≥III, n (%) | 3 (27.3) | 3 (27.3) | 1.000 |
| Histologic grade ≥2, n (%) | 5 (45.5) | 1 (9.09) | 0.056 |
| Lymphatic invasion, n (%) | 6 (54.6) | 6 (54.6) | 1.000 |
| Vascular invasion, n (%) | 2 (18.2) | 1 (9.09) | 0.531 |
| Perineural invasion, n (%) | 0 (0.0) | 1 (9.09) | 0.305 |

HLA, human leukocyte antigen; CEA, carcinoembryonic antigen; CA19-9, carbohydrate antigen 19-9; UICC, Union for International Cancer Control.
