# Supplementary material for: The prognostic impact of programmed cell death ligand 1 and human leukocyte antigen class I in pancreatic cancer
Source: Cancer Med. 2017 Jun 10;6(7):1614–26. doi: 10.1002/cam4.1087 (PMC5504334; doi:10.1002/cam4.1087)
Supplement: Supplementary file 11 — Table S3. Baseline characteristics of PDA patients with negative or positive PD‐L1 expression who underwent pancreatic resection. [file CAM4-6-1614-s011.docx]

**Supplementary Table S3.** Baseline characteristics of PDA patients with negative or positive PD-L1 expression who underwent pancreatic resection

| Factors | PD-L1 | | *p*-value |
| --- | --- | --- | --- |
|  | Negative (n=25) | Positive (n=11) |  |
| Gender, male (%) | 17 (68.0) | 6 (54.6) | 0.475 |
| Age (years) | 67 ± 2 | 70 ± 3 | 0.453 |
| CEA (ng/ml) | 4.3 ± 1.6 | 8.9 ± 2.8 | 0.177 |
| CA19-9 (U/ml) | 384 ± 236 | 716 ± 377 | 0.460 |
| Tumor size (cm) | 3.2 ± 0.2 | 3.0 ± 0.4 | 0.671 |
| pT4, n (%) | 9 (36.0) | 2 (18.2) | 0.439 |
| pN1, n (%) | 19 (76.0) | 9 (90.9) | 0.400 |
| UICC staging ≥III, n (%) | 10 (40.0) | 2 (18.2) | 0.268 |
| Histological grade ≥2, n (%) | 5 (20.0) | 6 (54.6) | 0.038 |
| Lymphatic invasion, n (%) | 17 (56.0) | 7 (63.6) | 0.445 |
| Vascular invasion, n (%) | 7 (29.2) | 1 (10.0) | 0.386 |
| Perineural invasion, n (%) | 5 (20.0) | 0 (0) | 0.293 |

HLA, human leukocyte antigen; CEA, carcinoembryonic antigen; CA19-9, carbohydrate antigen 19-9; PD-L1, programmed death ligand 1; UICC, Union for International Cancer Control.
