# Supplementary material for: The prognostic impact of programmed cell death ligand 1 and human leukocyte antigen class I in pancreatic cancer
Source: Cancer Med. 2017 Jun 10;6(7):1614–26. doi: 10.1002/cam4.1087 (PMC5504334; doi:10.1002/cam4.1087)
Supplement: Supplementary file 12 — Table S4. Baseline characteristics of PDA patients with negative or positive membranous PD‐L1 expression who underwent pancreatic resection. [file CAM4-6-1614-s012.docx]

**Supplementary Table S4.** Baseline characteristics of PDA patients with negative or positive *membranous* PD-L1 expression who underwent pancreatic resection

| Factors | PD-L1 | | *p*-value |
| --- | --- | --- | --- |
|  | Negative (n=30) | Positive (n=6) |  |
| Sex, male (%) | 20 (66.7) | 3 (50.0) | 0.438 |
| Age (years) | 67 ± 2 | 70 ± 4 | 0.648 |
| CEA (ng/ml) | 5.8 ± 1.6 | 3.0 ± 3.8 | 0.495 |
| CA19-9 (U/ml) | 555 ± 216 | 54.7 ± 503 | 0.368 |
| Tumor size (cm) | 3.2 ± 0.2 | 3.0 ± 0.4 | 0.671 |
| pT4, n (%) | 11 (36.7) | 0 (0) | 0.439 |
| pN1, n (%) | 23 (76.7) | 6 (100) | 0.187 |
| UICC staging ≥III, n (%) | 12 (40.0) | 0 (0) | 0.058 |
| Histological grade ≥2, n (%) | 7 (23.3) | 4 (66.7) | 0.035 |
| Lymphatic invasion, n (%) | 16 (55.2) | 5 (83.3) | 0.200 |
| Vascular invasion, n (%) | 8 (28.6) | 0 (0) | 0.134 |
| Perineural invasion, n (%) | 5 (16.7) | 0 (0) | 0.314 |

HLA, human leukocyte antigen; CEA, carcinoembryonic antigen; CA19-9, carbohydrate antigen 19-9; PD-L1, programmed death ligand 1; UICC, Union for International Cancer Control.
