# Supplementary material for: The prognostic impact of programmed cell death ligand 1 and human leukocyte antigen class I in pancreatic cancer
Source: Cancer Med. 2017 Jun 10;6(7):1614–26. doi: 10.1002/cam4.1087 (PMC5504334; doi:10.1002/cam4.1087)
Supplement: Supplementary file 13 — Data S1. Immunohistochemical staining. [file CAM4-6-1614-s013.docx]

**Supplementary Material and Methods**

**Immunohistochemical staining**

Formalin-fixed, paraffin-embedded tumor sections were assessed immunohistochemically. Briefly, 4-μm sections were deparaffinized in xylene and dehydrated in ethanol series. For antigen retrieval, the specimens were pretreated in a microwave for HLA class I (650 W, 10 min; 200 W, 5 min, in target retrieval solution, pH 6.0 (Dako, Glostrup, Denmark)), and in an autoclave (120 °C, 15 min) for PD-L1 in 0.5 M EDTA buffer; pH 8.0, for CD4, CD8, CD56 and CD68 in Target Retrieval solution, pH 9.0 (Dako, Glostrup, Denmark) and for PD-1 and FoxP3 in Target Retrieval solution, pH 6.0 (Dako, Glostrup, Denmark). The sections were incubated for 30 min in 0.3 % hydrogen peroxidase in absolute methanol to deactivate endogenous peroxidases. After blocking nonspecific binding of antibodies, the specimens were incubated with primary antibodies at room temperature for 30 min or at 4℃ overnight. Immunohistochemical staining was performed using an EnVision system and DAB kits (Dako, Glostrup, Denmark).
